# Comparison of psychological symptoms between infected and non-infected COVID-19 health care workers

**DOI:** 10.1186/s12888-021-03173-7

**Published:** 2021-03-26

**Authors:** Nami Mohammadian Khonsari, Gita Shafiee, Atefeh Zandifar, Sahar Mohammad Poornami, Hanieh-Sadat Ejtahed, Hamid Asayesh, Mostafa Qorbani

**Affiliations:** 1 Student Research Committee, Alborz University of Medical Sciences, Karaj, Iran; 2 Chronic Diseases Research Center, Endocrinology and Metabolism Population Sciences Institute, Endocrinology and Metabolism Research Institute, Tehran University of Medical Sciences, Tehran, Iran; 3 Non-communicable Diseases Research Center, Alborz University of Medical Sciences, Karaj, Iran; 4 Obesity and Eating Habits Research Center, Endocrinology and Metabolism Clinical Sciences Institute, Tehran University of Medical Sciences, Tehran, Iran; 5 Endocrinology and Metabolism Research Center, Endocrinology and Metabolism Clinical Sciences Institute, Tehran University of Medical Sciences, Tehran, Iran; 6 Department of Medical Emergencies, Qom University of Medical Sciences, Qom, Iran; 7 Social Determinants of Health Research Center, Alborz University of Medical Sciences, Karaj, Iran

**Keywords:** Health care workers, COVID-19, PTSD, Depression, Anxiety, Stress

## Abstract

**Background:**

Studies have shown that health care workers (HCWs), as front liners of the coronavirus (COVID-19) pandemic, are at high risk for psychological symptoms, but few studies have compared these symptoms in infected and non-infected HCWs. This study compares psychological symptoms among these two groups.

**Methods:**

In this cross-sectional study, 938 HCWs from various medical fields working in the leading general hospitals of Alborz province, Iran, were selected using a multistage sampling method. The participants had contact with COVID-19 patients. Post-traumatic stress disorder-8 (PTSD-8) is a validated questionnaire that we used to evaluate PTSD symptoms along with its subscales, including intrusion, avoidance, and hypervigilance. Also, the Depression, Anxiety, and Stress Scale-21 questionnaire was used to assess the severity of the aforementioned conditions in HCWs. Multivariate logistic regression was used to compare psychological symptoms in infected and non-infected HCWs.

**Results:**

Among 938 included HCWs, 55 had a history of confirmed COVID-19 infection. Prevalence of stress, anxiety, depression, intrusion, hypervigilance, and avoidance among infected HCWs were significantly higher in comparison to non-infected HCWs. In the multivariate logistic model, history of COVID-19 infection among HCWs was associated with a significantly increased risk of anxiety, depression, stress, intrusion, hyper-vigilance, and avoidance.

**Conclusion:**

The present study showed that the HCWs with COVID-19 infection were at a high risk of displaying psychological symptoms. Therefore, it is also necessary to develop psychological support and interventions for HCWs, especially those who got infected with the virus.

## Significant outcomes

It should be noted that not only the fear of infection but the disease itself may result in mental disturbances. HCWs as the front liners are in a very high risk of such mental disturbances. HCWs show signs of PTSD, depression, stress and anxiety; thus to help the HCWs to fully recover and provide their services as before, mental assessment and support are essential. We should keep in mind that the recovery from COVID-19 infection includes mental recovery as well.

## Limitations

As of our limitations, the results were limited by the number of participants. However, the most significant limitations were the process of finding willing participants and lacking the possibility of obtaining the history of previous psychiatric disorders in the participants. Other noteworthy limitations include the lack of sufficient funds and support.

## Introduction

The late coronavirus pandemic gave rise to many catastrophic events. Till now, January second, 2021, more than 82,579,768 got infected, and more than 1,818,849 have died [1]. In the first days of the outbreak of the virus, the main concerns included financial and human resources, hospital beds, and drug shortages [2]. One other problem that arose from the limited resources was the lack of an adequate number of physicians and health care workers (HCWs) that led to the recruitment of HCWs without pandemic training in the field of coronavirus disease-19 (COVID-19) [3]. This inevitable situation made everyone question the ability to manage these difficult conditions in these dire times [4]. In addition to the state of mental health among the general population, who may experience stress, anxiety, depression, and other related disorders, it is imperative to address the mental health status of the HCWs caring for patients with COVID-19 [5]. Many studies have illuminated the extent of the psychological damage done upon the family members and these healthcare workers [5, 6]. More recent studies elucidated that not only those who care for the sick or suffer a loss can develop psychological symptoms, but those who were sick can develop psychological symptoms [7]. The severity of the disease and the anxiety resulted from the smallest chances of one presuming to have acquired this condition has caused some degrees of mental distress [7, 8]. Although the severity of the disease may not be as such as people assume, due to the highlighting of severe cases and the news concerning the mortality rates, a horrific image of this virus has been made in the thoughts of the general population all around the world [9–11]. As much as difficult, the endurance of the mental burden of the disease may overwhelm the general population; it can be much more overwhelming for HCWs. The unimaginable workload due to the pandemic and work-related stress have worn out the health care system and caused mental distress for the HCWs [5, 7, 12–14]. Several studies show that HCWs as the front-line of the COVID-19 pandemic are at high risk for developing psychological symptoms such as stress, anxiety and depression, due to their vital role in management of patients and the higher probability of infection; However to our knowledge no studies have compared these symptoms in infected and non-infected HCWs [7, 15].

### Current study

This study aims to evaluate and compare psychological symptoms among infected and non-infected health care workers as we suspect infected HCWs will have worse mental health outcomes than non-infected professionals, and provide a better understanding of the mental disturbances that may follow after one’s infection, and assess the vulnerable psychological domains to provide better care after infection.

## Methods

### Setting

This study took place in April and May 2020 on HCWs of various wards of nine of the main hospitals admitting COVID-19 infected patients in Alborz province of Iran with a population near to three million. This province is one of the most populated provinces of Iran, with insufficient numbers of HCWs for patients with COVID-19.

### Sample size and participants

The present study is based on data obtained from the previously published study. The sample size was calculated approximately 940 subjects by considering type I error, the prevalence of psychiatric distress, and precision as 5, 29, and 2.9%. This multi-center cross-sectional study included 938 randomly selected HCWs. Participants were HCWs who were in contact (direct or indirect) with COVID-19 patients within various hospital wards. Participants were selected via a proportional random sampling method. Sampling within each hospital was conducted according to the number of HCWs in each hospital, proportional to the number of HCWs in the hospital. These HCWs were comprised of physicians, nurses, and hospital technicians. The data used in this study was collected and evaluated electronically via the university’s website in which the participant would fill out the online questionnaire. The study was completely optional, and only those who wished to participate in the study entered. The participants were HCWs who wished to enter the study from various wards of the leading hospitals of Alborz University of Medical Sciences in Alborz Province, Iran. At the beginning of the questionnaire, there was a statement regarding whether the participant would consent to the use of their data for research purposes and publication. All those questionnaires with an electronically signed consent or checked consent option in the questionnaire were included in this study.

### Data collection

Participants filled the demographic and work-related characteristics questionnaire, including age, gender, and education, and occupation, type of employment (temporary, permanent, or medical resident), type of ward, and job duration. HCWs had direct contact, such as HCWs in emergency, infection, and ICU wards were considered as front-line staff, and HCWs in other ward were considered as non-front-line staff. Demographic and work-related characteristics were missing in some participants. Their psychological status was assessed by the Depression, Anxiety, and Stress Scale-21 (DASS-21) questionnaire. Furthermore, post-traumatic stress disorder questionnaire 8 (PTSD-8) was used to assess PTSD symptoms. In this study, a 21-item DASS (DASS-21) questionnaire was used, consisting of seven questions for each of the depression, anxiety, and stress domains. Participants filling out the form can choose one of the four choices that were used for scoring from zero to three for each question. The total score that is obtained from the sum of all scores indicates the severity of depression, anxiety, or stress, with higher scores related to more severe cases [16]. The maxim achievable score in each domain was 21. The cut-off values for stress, anxiety, and depression, are 14, 9, and 7, respectively. Some example items from the DASS-21 include questions regarding relaxation and agitation [16]. The reliability and validity of this questionnaire among the Iranian population have been evaluated, indicating excellent consistency and good test-retest reliability with acceptable concurrent validity in all three sub-scales. In Iranian studies by Samani et al. and Asghari et al., Cronbach’s alpha for depression, anxiety, and stress was .93, .90, and .92, respectively. Thus this questionnaire can be used in the adult Iranian population with satisfactory psychometric properties, with the same cut-off values [17, 18].

PTSD-8 is comprised of 8 items, which corresponds to the DSM-IV criteria for PTSD [19]. The eight PTSD items are divided into three subscales that correspond to the three main symptom groups: intrusion (4 items), avoidance (2 items), and hypervigilance (2 items). Each item is responded on a four-point Likert scale (‘not at all’ (1), ‘a little’ (2), ‘quite a bit (3), and ‘very often’ (4)). In each subscale, if at least one item, the score was equal to or greater than three (≥ 3) was considered as positive in that PTSD subscale. Some example items from the PTSD-8 questionnaire include questions regarding sleeping difficulty, Irritability and Difficulty concentrating [20]. This straightforward and short questionnaire has high sensitivity and specificity. The questionnaire was validated by Hansen et al. [20]. In three different trauma samples. The Cronbach’s alpha in the Iranian population for these three samples are .82, .81, and .68, respectively, indicating the sufficiency and acceptability of the use of this questionnaire in the Iranian population [21]. With the aid of this questionnaire, we made a proper assessment of the status of three related domains of PTSD (intrusion as of reliving the traumatic event all over again, avoidance as of attempting to avoid the distressing memories, thoughts, and feelings as well as external reminders regarding the traumatic event, and hyper-vigilance as of the state of extreme alertness that decreases the quality of life). All participants were asked if they had signs or symptoms of COVID-19 infection and if they have been tested with polymerase chain reaction (PCR), and all those with positive PCR results were presumed to have had COVID-19 infection.

### Statistical analysis

We used SPSS software (made by International Business Machines Corporation (IBM). USA) version 16 for data analysis. Demographic, work-related characteristics and psychological symptoms was not filled by some participants. The normal distribution of continuous variables was assessed using the Kolmogorov-Smirnov test. Continuous variables with and without normal distribution were represented as mean (standard deviation (SD)) and median (interquartile range (IQR)), respectively. Qualitative variables were expressed as frequencies and percentages. The frequency of categorical variables such as age categories, gender, being front-line staff, type of employment, occupation, and education, and job duration categories among infected and non-infected HCWs was compared using the Chi-square test. The Mann-Whitney U was used to compare the Median (IQR) of total and subscale psychological symptoms (PTSD and DASS-21) score among infected and non-infected HCWs. Spearman’s correlation coefficient was used to assess the correlation between total and subscale of PTSD and DASS score in infected and non-infected HCWs. All *p*-values of Spearman’s correlation were corrected using the Benjamini-Hochberg correction method to control the multiple comparisons problem [22]. The association of infection with COVID-19 (as the independent variable) with psychological symptoms **(**depression, anxiety, stress, intrusion, avoidance, and hyper-vigilance) (as dependent variables) was assessed using univariate and multivariate logistic regression model. In the multivariate model, all demographic characteristics according to empirical evidence and theoretical knowledge of confounding factors were included in the model [23, 24]. The results of logistic regression analysis were presented as odds ratio (OR) and 95% confidence interval (CI).). A *p*-value less than 0.05 was considered as statistically significant (*p*-value < 0.05).

### Ethical considerations

This study was approved by the ethics committee and ethics in the research of Alborz University of Medical Sciences of Iran. We carefully explained the study to our participants, and all the aspects, components, and objectives of the project were illuminated in the online questionnaire upon entering the study. All participants could withdraw from the study at any point with no penalty. The compensation of the participants was not applicable to this study. All methods were carried out under relevant guidelines and regulations. Informed consent was obtained from all subjects, and since all subjects were above eighteen, no consent from legal guardians was needed. At the end of the online evaluation questionnaire, the participants had the option to send contact information for further mental assessment, clinical advice, and psychological treatment if necessary; all those in need of these assists were provided, free of charge, by our expert team of psychiatrists and psychologists.

### Data availability statement

We took all components of Helsinki law into account in this project. We gave assurance to our participants that all information obtained from them shall be kept confidential and will not be shared with any individual, group, or organization. However, upon reasonable request, and with the consent of the participants, the data without any personal information that may lead to breaking one’s confidentiality can be given to be assessed for authenticity purposes. The data can be obtained from the corresponding or the first author.

## Results

Overall, 938 personnel participated in our study, 55 of whom had a history of confirmed COVID-19 infection comprising 19 males and 35 females. As shown in Table 1, the frequency of permanent HCWs in the infected group was significantly higher than the non-infected group (x^2^: 6.72, *P*-value: 0.035). Moreover, the association of occupation and COVID-19 infection was statistically significant (x^2^: 8.70, *P*-value: 0.013). The infected and non-infected HCWs did not differ significantly in terms of other demographic and work-related characteristics (*P*-value> 0.05).

**Table 1:** Demographic and disease related characteristics in infected and non-infected HCWs with COVID-19

variable		Infected HWCs		Non-infected HCWCs		x^2^ statistic	*P-*value
		N	%	N	%		
Age(year)	< 30	15	27	281	34	1.93	0.38
	31–40	23	42	351	43		
	> 40	17	31	193	23		
Gender	Male	19	35	235	28	1.27	0.26
	Female	35	65	603	72		
Frontline staff	No	23	42	452	54	3.08	0.08
	Yes	32	58	387	46		
Type of employment	Permanent	34	62	367	44	6.72	0.035*
	Temporary	20	36	423	51		
	Medical resident	1	2	40	5		
Education	= < diploma	6	11	118	14	0.65	0.72
	Bachelor	36	67	552	67		
	> Bachelor	12	22	156	19		
Occupation	Physician	7	13	73	9	8.70	0. 013*
	Nurse staff	40	75	503	61		
	Technician	6	11	247	30		
Job duration (year)	< 5	35	70	467	78	2.63	0.26
	6 to 10	12	24	91	15		
	> 10	3	6	38	7		

*Statistically significant

*HCWs* Health Care Workers

*P-*value based on Chi-square test

In Table 2, the median of DASS and PTSD subscales score was compared in the infected and non-infected HCWs. All Depression, anxiety, stress, intrusion, hypervigilance, avoidance, and PTSD-8 are significantly more common in those with a history of COVID-19 infection in comparison to those who never got infected (*p*-value < .001).

**Table 2:** Median (IQR) of total and subscale psychological symptoms score among infected and non-infected HCWs

Psychological symptoms	Non-Infected HWCs		Infected HWCs		Mann-Whitney U statistics	*p-*value
	Median	IQR	Median	IQR		
Depression score	8	12	16	12	−4.68	< 0.001*
Anxiety core	8	12	16	14	−5.64	< 0.001*
Stress score	10	14	18	10	−4.27	< 0.001*
Avoidance score	7	3	8	4	−3.34	0.001*
Hyper-vigilance score	4	3	5	2	−4.98	< 0.001*
PTSD total score	4	3	5	2	−5.22	< 0.001*

*PTSD* Post traumatic stress disorder, *IQR* Inter-quartile range, *HCWs* Health Care Workers

*Statistically significant

*P-*value based on Mann-Whitney U test

Correlations between all DASS-21 subscales and PTSD-8 subscales in both of the infected and non-infected groups were observed in Table 3. The correlations were more substantial in the non-infected group. Nonetheless, strong correlations can also be seen in the infected group.

**Table 3:** Correlation between total and subscale psychological symptoms score among infected and non-infected HCWs

Infected HCWs							
	Depression score	Anxiety score	Stress score	PTSD total score	Intrusionscore	Avoidance score	Hyper-vigilance score
Depression score	1	–	–	–	–	–	–
Anxiety score	0.76*	1	–	–	–	–	–
Stress score	0.81*	0.81*	1	–	–	.-	–
PTSD total score	0.55*	0.56*	0.58*	1	–	–	–
Intrusion score	0.46*	0.47*	0.49*	0.91*	1	–	–
Avoidance score	0.48*	0.50*	0.51*	0.84*	0.65*	1	–
Hyper-vigilance score	0.50*	0.52*	0.53*	0.81*	0.62*	0.60*	1
Non-Infected HCWs							
Depression score	1	0.79*	0.88*	0.78*	0.68*	0.58*	0.67*
Anxiety score	–	1	0.87*	0.72*	0.57*	0.58*	0.77*
Stress score	–	–	1	0.76*	0.63*	0.58*	0.78*
PTSD total score	–	–	–	1	0.90*	0.80*	0.83*
Intrusion score	–	–	–	–	1	0.58*	0.61*
Avoidance score	–	–	–	–	–	1	0.67*
Hyper-vigilance score	–	–	–	–	.-	–	1

*PTSD* Post traumatic stress disorder, *HCWs* Health care workers, *DASS-21* the Depression, Anxiety, and Stress Scale-21

Values are Spearman correlation coefficient

*Statistically significant (*p*-value < 0.05) and are corrected with the Benjamini-Hochberg correction method

**Fig. 1:**
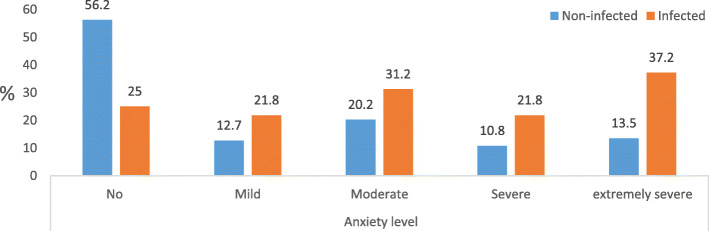
Anxiety level according to infection status. Number of Infected patients: 55. Number of Non-infected patients: 884

**Fig. 2:**
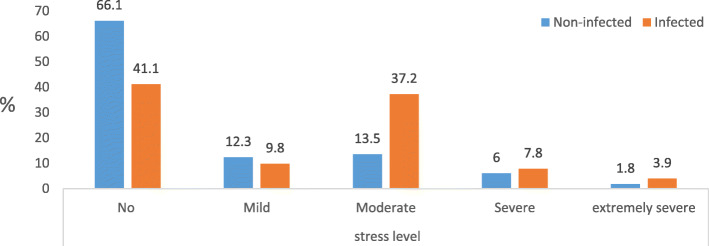
DASS-21 stress level according to infection status. Number of Infected patients: 55. Number of Non-infected patients: 884

**Fig. 3:**
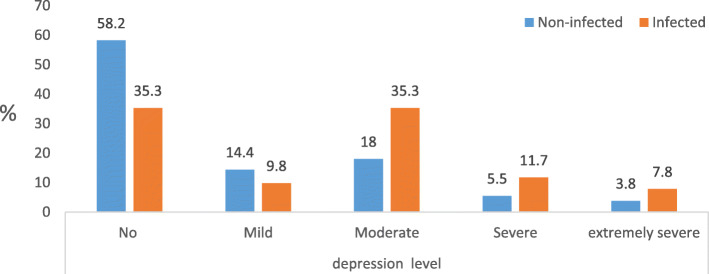
Prevalence of DASS-21 depression level according to infection status. Number of Infected patients: 55. Number of Non-infected patients: 884

The prevalence of all DASS-21 subscales and PTSD-8 subscales are presented in Table 4. All DASS-21 subscales and PTSD-8 subscales in infected HCWs were significantly higher than their non-infected counterparts (*p* < .05). The prevalence of stress, anxiety, and depression severity among infected and non-infected HCWs are illustrated in Figs. 1, 2, 3. As can be seen, the prevalence of more severe forms of anxiety, stress, and depression was much higher among infected HCWs, whereas mild to no signs of mental conditions were observed in the non-infected group.

**Table 4:** Association of infection with COVID-19 and psychological symptoms in logistic regression analysis

Psychological symptoms	Prevalence in Infected / Non-infected	Model I	Model II
	%	OR(95% CI)	OR(95% CI)
Intrusion^a^	66/43	2.25 (1.17–4.32)*	2.49 (1.27–4.85)*
Avoidance^a^	59/30	2.93 (1.58–5.41)*	3.25 (1.73–6.11)*
Hyper-vigilance^a^	67/36	3.36 (1.76–6.41)*	3.71 (1.91–7.20)*
Depression^b^	67/42	3.28 (1.67–6.43)*	3.74 (1.86–7.52)*
Anxiety^b^	85/51	4.61 (2.0–10.6)*	5.83 (2.38–14.29)*
Stress^b^	60/40	2.86 (1.50–5.47)*	3.34 (1.71–6.51)*

^a^Intrusion, avoidance and hyper-vigilance were defined score ≥ 3 according to PTSD-8 questionnaire

^b^Depression, anxiety and stress were defined score more than 14, 9, and 7 respectively according to DASS-21

Model I: Crude model

Model II: Adjusted for age, gender, occupation, type of employment, work in front-line, job duration, education

*statistically significant (*p*-value < .05)

*P*-value based on logistic regression analysis (psychological symptoms are dependent variables and COVID-19 infection is independent)

Number of Infected patients: 55

Number of Non-infected patients: 884

In a multivariate logistic regression model, history of COVID-19 infection among HCWs was associated with significantly higher levels of anxiety, depression, stress, intrusion, hyper-vigilance, and avoidance; these results are summarized in Table 4.

## Discussion

Although some studies have evaluated the mental health impact of the COVID-19 infection on various groups and populations, to the best of our knowledge, the current study is one of the first to compare psychological symptoms in infected and non-infected COVID-19 HCWs. Nonetheless, some studies evaluated similar aspects of psychological conditions evaluated in this study. These studies had concordant results with the current study and suggested that the COVID-19 pandemic had psychological effects on HCWs [25, 26]. The present study showed that HCWs infected with COVID-19 had significantly higher levels of depression, anxiety, and stress. Our results showed that the risk of psychological symptoms such as anxiety, stress, depression, intrusion, hypervigilance and avoidance was higher in infected HCWs with COVID-19 disease.

The rapid spread of the COVID-19 in the world and the existence of asymptomatic carriers make many challenges for healthcare systems in countries. In addition to the above issues, the high prevalence of the disease, lack of medication and protective agents because of sanctions and economic problems create many problems for controlling the disease in Iran.

COVID-19, similar to other epidemics and contamination outbreaks of diseases, has been followed by public health concerns and psychological impacts in the general population and also among the medical staff. HCWs, due to the critical situation in the COVID-19 pandemic are at risk of developing psychological problems. Some factors such as the fear of infection of themselves and family members, work overload, the lack of effective treatment, death of colleagues and family members and etc., can take a negative impact on mental health [27]. These factors cause a stress reaction to mental and physical health and also on interpersonal relationships [28]. A recent systematic review showed that delirium was a common sign in the acute phase of SARS and MERS, and COVID-19 [29]. Other common findings were depression, anxiety, insomnia, and PTSD [29]. Medical staff, as front liners of this pandemic, are under pressure due to the anxiety and stress; because of their vital role in the management of patients and the risk of acquiring the infection. Therefore, they are more at risk of developing mental distress symptoms [29].

However, personal reactions in a disaster will be different. Some people could improve their lives’ function and return to their previous life. Nevertheless, others retained their previous symptoms. These psychological adjustments related to individual differences, such as personality traits, interpersonal support systems, and the ability to cope with stress, may have played a major role and likely influenced the prognosis [30].

Several studies showed the effect of the COVID-19 outbreak on the mental health of HCWs [31, 32]. A large study in China focused on 2250 healthcare workers to assess mental health by measuring symptoms of anxiety, depression, and insomnia. The findings of this study showed that healthcare workers had symptoms of anxiety (34.7%), depression (19.8%), and poor sleep (23.6%) [31]. Another study found that HCWs experienced symptoms of distress (71.5%), depression (50%), anxiety (44.6%), and insomnia (34%) [33]. Also, in our study, a significant proportion of HCWs showed anxiety and depression symptoms.

Previous studies showed that in infectious diseases outbreak, the prevalence of stress, anxiety, depression, and other psychological symptoms in front-line medical workers in emergency, infection, and ICU wards who had direct contact with infectious patients, were more than HCWs in other wards [34–36].

A very important group at risk for psychological symptoms are the medical staff who are infected with COVID-19. These patients were exposed to an increased burden of stress, and they had the most negative psychological responses of any group. To our best knowledge, this is one of the first studies comparing the psychological symptoms between infected HCWs and uninfected HCWs during the first peak of the COVID-19 pandemic in April and May 2020 in Iran. Our results showed that infected HCWs had more severe symptoms of depression, anxiety, and distress. Also, the prevalence of other disorders, such as PTSD, was higher in infected HCWs. Multivariate logistic regression analyses showed that COVID-19 infection was associated with psychological symptoms after adjustments for potential confounders such as age, gender, type of employment, education, job duration and being a front liner. Many factors that have been reported to be associated with the risk of psychological symptoms in COVID-19 disease are already known to be risk factors for mental health conditions. The socio-demographic factors such as lower education, living alone, females, older people, and having a medical history, especially psychiatric disorders, were associated with depression and/or anxiety symptoms [37, 38]. However, these factors are not the only important risk factors of psychological symptoms and disorders*,* job-related factors such as working in front-line compared to the second line, > 10 years of working and especially infected HCWs are relevant, newly added established risk factors associated with increased risk of developing psychological symptoms in this pandemic [33, 34].

Previous studies have found that a long period in which front-line HCWs continued to be exposed to a disaster such as COVID-19 or SARS could increase PTSD and other psychological symptoms; in these studies, High PTSD levels among hospital workers persisted over a long period of time [39, 40]. The fear of transition of the disease to family or friends, death, being quarantined, and fear of the recurrence of COVID-19 could increase PTSD and other psychological symptoms among infected HCWs. In this regard, our findings show that infected HCWs had significantly more depression, anxiety, stress, avoidance, and PTSD.

Although the government has done several activities to prevent the further spread of the COVID-19 outbreak in the community, there are still no targeted interventions to reduce the psychological burden. The governmental organizations should further publish correct information about the mental health burden among the people and high-risk population. Also, a uniform mental health counseling platform to provide psychological counseling should be established. Particular attention should be paid to HCWs mental health; especially, those who have been in contact with patients or quarantined people or are infected with COVID-19. In addition, a balance between work and rest, doing other activities such as physical activity and exercise, and promoting sleep quality is the need for HCWs.

There are some limitations to our study. First, it was a cross-sectional study; it is impossible to inference any cause-effect relationships. Second, all participants were selected from one city, and our findings could not generalize to the entire country. Third, in this study, information about the workload of HCWs, quarantine, and its duration was not asked from the participants, which may affect our findings. Finally the most critical limitation was the lacking of the possibility of obtaining the history of previous psychiatric disorders in the participants.

## Conclusion

In this study, we identified that the HCWs with COVID-19 infection were at a high risk of displaying psychological symptoms. Therefore, it is also necessary to develop psychological support and interventions for HCWs, especially those who got infected with the virus.

Despite all rational expectations that those who got infected and recovered may have a calmer state of mind, feeling that they have beaten the disease and they may not get sick anymore, in this study we found a much higher prevalence of severe forms of psychological symptoms among the infected yet recovered HCWs in comparison to non-infected HCWs. These findings show how detrimental being infected can be on HCWs, psychologically, resulting in high levels of psychological symptoms. HCWs are the most prone to this infection, and this pandemic has resulted in much hardship for them. Contracting the infection can result in PTSD and severe psychological symptoms, and thus we recommend that psychiatric consulting may be beneficial for those who have recovered from the COVID-19 infection, specially HCWs who seem to need this counseling the most to prevent further psychological symptoms and aid to their complete recovery.

## Data Availability

We reassured our participants that all information obtained from them shall be kept confidential and not shared with any individual, group, or organization. However, upon reasonable request, and with the participants’ consent, the data without any personal information that may lead to breaking one’s confidentiality, are available from the corresponding or the first author. The data can be given to be assessed for authenticity and research purposes.
